# Satellite data is revealing long time changes in the world largest lakes

**DOI:** 10.1038/s41598-024-65250-7

**Published:** 2024-06-22

**Authors:** Tiit Kutser, Tuuli Soomets

**Affiliations:** Estonian Marine Institute, University of Tartu, Mäealuse 14, 12618 Tallinn, Estonia

**Keywords:** Large lakes, Remote sensing, Climate change, Carbon cycle, Water quality, Aquatic optics, Limnology, Freshwater ecology, Climate-change ecology

## Abstract

Lakes are a crucial source of drinking water, provide ecological services from fisheries and aquaculture to tourism and are also a critical part of the global carbon cycle. Therefore, it is important to understand how lakes are changing over time. The ESA Ocean Colour Climate Change Initiative (OC-CCI) database allows to study changes in the largest lakes over 1997–2023 period. The Caspian Sea and ten next largest lakes were under investigation. Changes in the phytoplankton biomass (Chl-a), the concentration of particulate matter (*b*_bp_(555)), the colored dissolved organic matter, CDOM (*a*_dg_(412)), and the light diffuse attenuation coefficient in water (*K*_d_(490)) were analyzed. Both increasing and decreasing trends (or no significant trend at all) of studied parameters were observed in these lakes over the study period. In some of the Laurentian Great Lakes the changes in CDOM over the study period were found to be in accordance with the lake water level changes i.e. with the inflow from the catchment. There was difference between the trends of Chl-a and *b*_bp_(555) in lakes Michigan and Huron indicating that there may have been shift in phytoplankton community that took place around 2005. The study demonstrated that remote sensing products, like the ones created by ESA OC-CCI, are valuable tools to study behavior of large lakes ecosystems over time.

## Introduction

Lakes play an important role in global biogeochemical cycles, contribute significantly to climate regulation, are a crucial source of drinking water, and provide significant number of ecological services from fisheries and aquaculture to tourism and recreation^1–3^. The studies on changes in lakes include trends in lake water temperature^4^ and primary production^5^, increase of phytoplankton blooms in lakes^6–8^ or altering of mixing regimes^9^. Nevertheless, Minor and Oyler^10^ showed that the dissolved organic carbon (DOC) and its coloured fraction CDOM are the most understudied components of the carbon cycle despite their relevance to the Earth’s biogeochemistry and climate^11^.

An increase of concentration of CDOM in natural waters is called browning. There are several studies that show browning of Boreal lakes^12–17^. Browning increases resources for heterotrophic microorganisms, decreases primary production due to lower light availability, and has negative impacts on higher trophic levels^2,18^. Browning increases surface water temperature^19^ as in CDOM-richer waters light is absorbed in thinner surface layer and consequently, the radiative heating takes place in thinner layer than in less absorbing waters. This has an impact also on lake mixing regimes^9^.

A very small fraction of the 117 million lakes on Earth ^20^ is monitored regularly and has long data archives. Even if collected then the in situ data does not have sufficient spatial and temporal coverage for large scale (e.g. climate) studies. The European Space Agency (ESA) has established Climate Change Initiative (CCI) the aim of which is to create consistent satellite data series that allow studies on trends in different climate related parameters. The CCIs take data from all available satellites and produce harmonized products like they would have been produced by a single sensor. Meaning that band shifts and other recalculations (due to different spatial resolutions, sensitivities, etc.) are made to be able to get a consistent reflectance spectra. The reflectance data is used to calculate different water quality products and these products are then validated to assess the consistency of products obtained from different satellite sensors. This allows to make longer time series of different satellite products and to study potential changes happening during this time period.

There are currently more than 20 CCI projects that cover topics like clouds, ozone, sea level, sea surface temperature, permafrost, etc. Lake CCI is a recent addition of the Initiative. It contains products like water level, water extent, surface water temperature, ice cover, ice thickness and water-leaving reflectance. However, the Lake CCI does not produce some critical parameters important from the global carbon cycle and climate change studies perspective, like CDOM concentration. Therefore, we decided to use Ocean Colour CCI (OC-CCI) products in this study. The latest version (v6) of the OC-CCI product is based on SeaWiFS, MERIS, Aqua-MODIS, VIIRS and OLCI satellite data and is recalculated to mimic OLCI data series.

Majority of the lakes on Earth are too small to be monitored with 4 km pixel size, which is the spatial resolution of the OC-CCI products. However, our study focuses on the Caspian Sea and the ten next largest lakes on Earth where the 4 km spatial resolution is adequate. Moreover, the Caspian Sea, the ten next largest lakes, and the rest of the 117 million lakes contain roughly one third of the global lake water volume each (30%, 30% and 40% respectively). Consequently, studying the Caspian Sea and the ten next largest lakes (the aim of this study) covers 60% from the global lake water volume.

The OC-CCI data contains different water quality parameters and the data series starts from September, 1997. We focused on studying changes in four key water quality parameters: 1) CDOM absorption coefficient (using light absorption coefficient at 412 nm product, *a*_dg_(412), as a proxy); 2) phytoplankton biomass (using OC-CCI Chl-a product as a proxy); 3) concentration of particulate matter (using particle backscattering at 555 nm product, *b*_bp_(555), as a proxy);) and 4) water transparency (using diffuse attenuation coefficient at 490 nm, *K*_d_(490), product). These parameters characterise the impact of different optically active substances on water colour and allow to assess what kind of changes (if any) are taking place in the World largest lakes. It must be noted that the *a*_dg_(412) product combines both absorption by CDOM and absorption by detrital matter that are hard to separate from each other due to nearly identical spectral feature (high absorption in the ultraviolet part of the spectrum decreasing exponentially towards longer wavelengths in the visible part of spectrum). Our aim was to find whether there is browning happening in the World largest lakes rather than mapping precise concentration of CDOM. Thus, from that point of view it is not critical whether the browning (if any) happens due to dissolved or particulate matter.

## Results

The Mann–Kendall seasonal test was conducted to all four parameters, in Table 1 the *z*-stats and the significance as* p*-value is shown for each lake. The number of z-stats shows the magnitude of the trend and positive number shows the increasing trend while negative value shows decreasing trend. The existence of the trend over the study period is shown with bold.

**Table 1:** *z*-stats and the significance (*p*-value) of the Mann–Kendall seasonal tests (alpha 0.05) for the colored dissolved organic matter, CDOM (*a*_dg_(412)), the phytoplankton biomass (Chl-a), the concentration of particulate matter (*b*_bp_(555)), and the diffuse light attenuation coefficient in water (*K*_d_(490)).

	a_dg_(412)		Chl-a		*b*_bp_(555)		*K*_d_(490)		*n*
	*z-*stats	*p*-value	*z-*stats	*p*-value	*z-*stats	*p*-value	*z-*stats	*p*-value	
*Caspian Sea*	− 0.01	0.9897	1.53	0.1261	0.07	0.9480	0.56	0.5730	313
*Great Bear L*	**4.94**	< **0.0001**	1.30	0.1947	**2.74**	**0.0062**	**2.09**	**0.0362**	132
*L. Baikal*	**2.59**	**0.0096**	− 0.28	0.7793	**2.86**	**0.0043**	1.91	0.0566	228
*L. Erie*	0.54	0.5886	− 0.90	0.3667	1.91	0.0561	− 0.32	0.7525	294
*L. Huron*	** − 3.62**	**0.0003**	** − 15.29**	< **0.0001**	** − 6.91**	< **0.0001**	** − 6.79**	< **0.0001**	312
*L. Malawi*	1.48	0.1379	**1.99**	**0.0469**	− 0.69	0.4877	− 0.35	0.7269	312
*L. Michigan*	** − 5.68**	< **0.0001**	** − 15.54**	< **0.0001**	** − 10.12**	< **0.0001**	** − 10.51**	< **0.0001**	311
*L. Ontario*	− 1.88	0.0598	** − 3.05**	**0.0023**	** − 2.26**	**0.0238**	** − 2.32**	**0.0205**	311
*L. Superior*	1.49	0.1354	**4.06**	< **0.0001**	0.38	0.7026	0.17	0.8639	309
*L. Tanganyika*	** − 1.99**	**0.0464**	− 1.47	0.1407	** − 3.43**	**0.0006**	− 3.87	0.0001	311
*L. Victoria*	** − 7.18**	< **0.0001**	** − 8.64**	< **0.0001**	** − 4.56**	< **0.0001**	− 9.20	< **0.0001**	311

The positive number of the z-stats indicates increasing trend, while negative marks decreasing trend. The significant trend is indicated with bold font.

**Figure 1:**
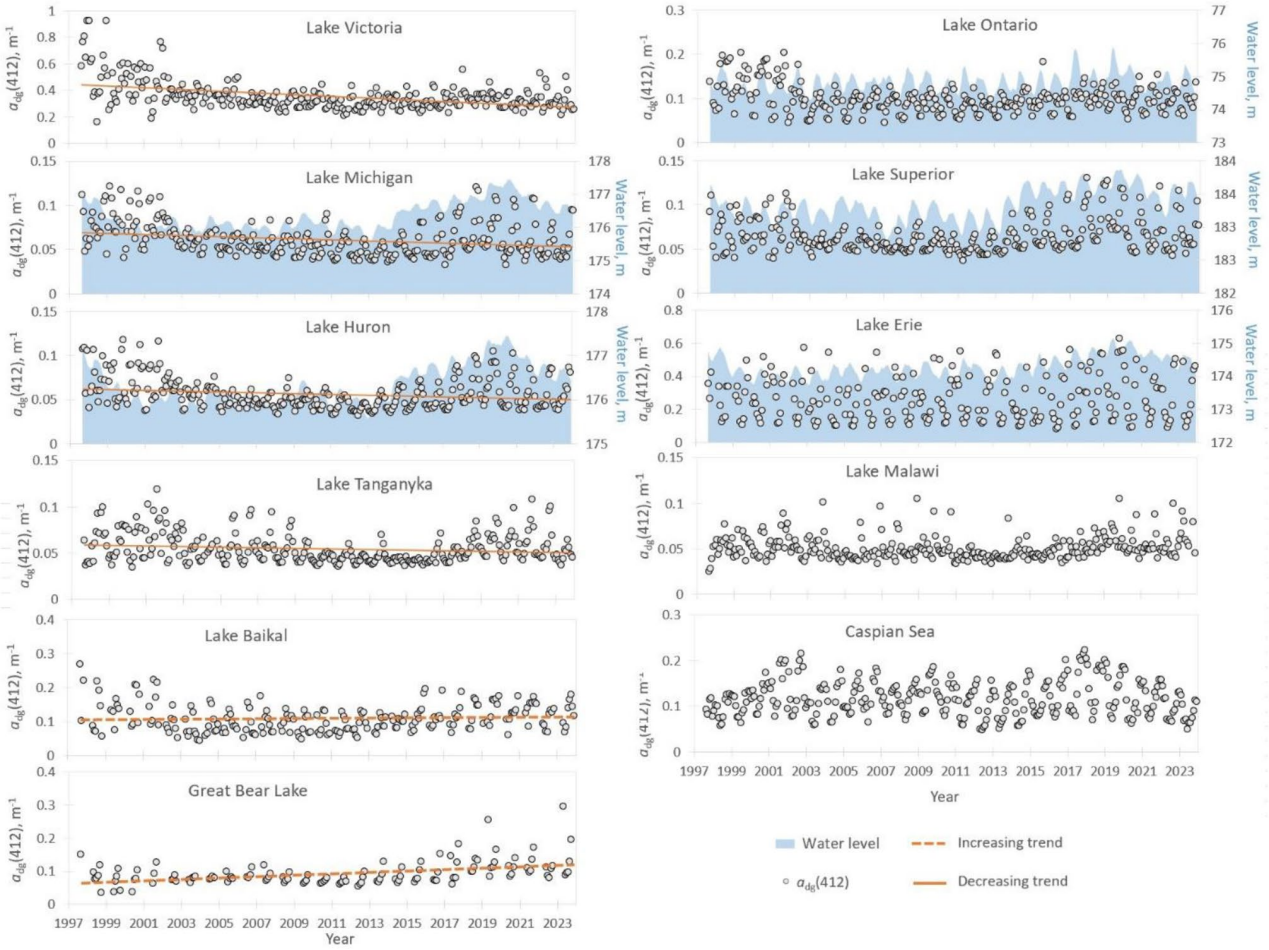
Variation of CDOM over the 1997–2023 study period. Monthly mean values (black circles) and trends of the OC-CCI *a*_dg_(412) product (a proxy of CDOM) in eleven of the World’s largest lakes. Water level in some of the Laurentian Great Lakes (NOAA GLERL database, Hunter et al. 2015) is shown in blue.

Monthly mean values of *a*_dg_(412) absorption coefficient are shown in the Fig. 1. Statistically significant positive or negative trends in the absorption coefficient time series were identified in the case of six lakes: L. Victoria, L. Michigan, L. Baikal, Great Bear L., L. Huron, and L. Tanganyika. However, it is seen in the case of all other lakes than L. Baikal and Great Bear L. that there has been decreasing trend in CDOM over the whole 1997–2023 time period. The most significant decrease has been in Lake Victoria. Yet, the largest increase has taken place in several Laurentian Great lakes during the last decade, despite of the decreasing trend over the whole study period. It is seen in the case of these lakes that the *a*_dg_(412) values were high in the end of 1990s and from around 2020, but there are lower monthly means between 2003 and 2013. Thus, even if a negative trend exists over the whole study period (Table 1), then we have to keep in mind that the actual changes have been more complicated than the long time trend. In the case of L. Tanganyika and the Caspian Sea one may observe even three periods with higher CDOM absorption followed by decrease.

**Figure 2:**
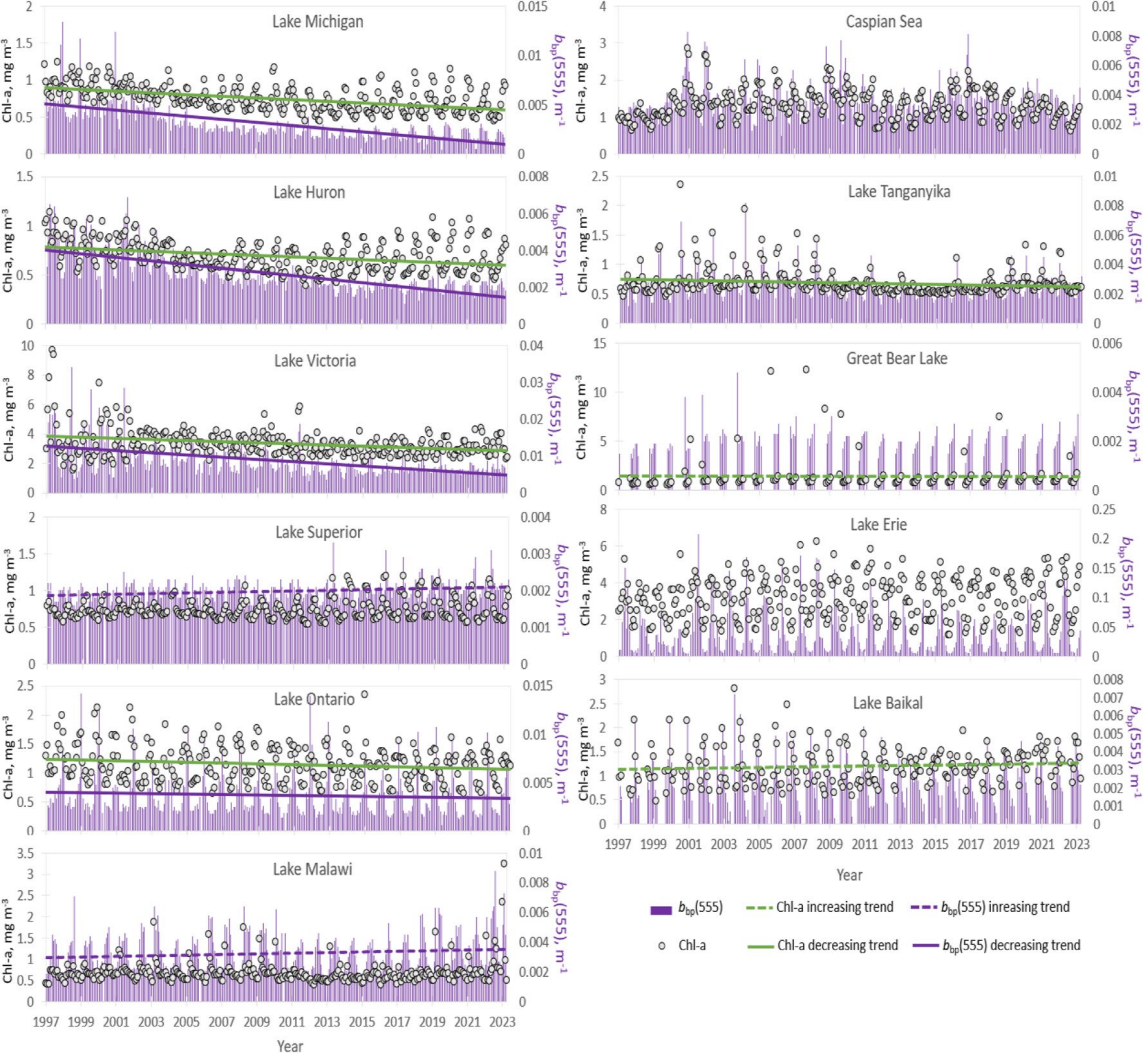
Variations of Chl-a and backscattering coefficient over the study period. Monthly mean values (black circles) and trends of OC-CCI Chl-a product and particle backscattering coefficient *b*_bp_(555) (purple bars) with trends in eleven World’s largest lakes over the 1997–2023 period.

Seven of the studied lakes had statistically significant positive or negative trend in phytoplankton biomass—Chl-a (Fig. 2). The decreasing trend was most obvious in L. Michigan, L. Huron and L. Victoria, while L. Baikal and Great Bear L. had increasing biomass during the study 1997–2023 study period. It must be noted that the biomass in L. Huron had a similar double peak like CDOM (and some other lakes), but in other lakes the behaviour of Chl-a was different from that of CDOM.

Several studied lakes showed decreasing trend in particulate matter backscattering coefficient *b*_bp_(555) (Fig. 2), for example L. Michigan, L. Huron, L. Ontario, and L. Victoria. On the other hand, the particulate matter backscattering was increasing during the study period in L. Superior, and L. Malawi. There was no significant trend in the other studied water bodies.

**Figure 3:**
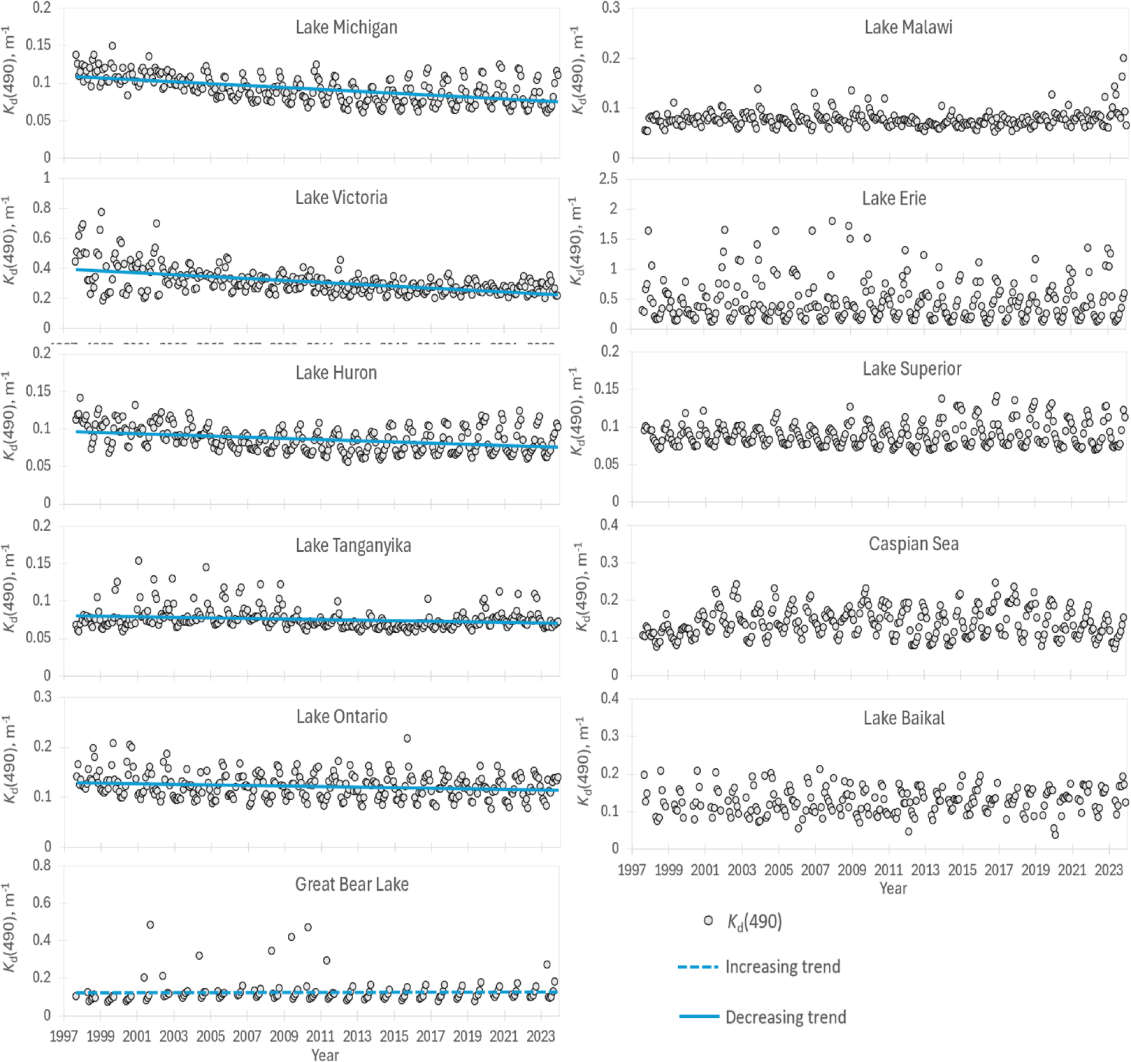
Variation of light attenuation coefficient over the study period. Monthly mean values (black circles) and trends of OC-CCI *K*_d_(490) product (a proxy of water transparency) in eleven World’s largest lakes over the 1997–2023 period.

The *K*_d_(490) (Fig. 3) describes the combined effect of light absorption and scattering processes in water. Trends in the *K*_d_(490) should be similar to the trends in the optically dominant water constituent. In large lakes and seas phytoplankton biomass drives the optical water properties as terrestrial CDOM and mineral particles do not travel far from land and the autochthonous CDOM and particulate matter in open parts of the large waterbodies are both phytoplankton degradation products. The light attenuation coefficient *K*_d_(490) decreased over the study period in L. Michigan, L. Victoria, L. Huron, L. Tanganyika, and L. Ontario. The Great Bear L. showed small increasing trend of *K*_d_(490). Other lakes did not show statistically significant trends.

## Discussion

The OC-CCI products have been designed for relatively clear marine waters. Therefore, the first step of the study was evaluating are the OC-CCI products suitable to study the World largest lakes. The OC-CCI products have been validated by the ESA OC-CCI project team for a certain range of optical properties and concentrations of optically active substances. The values of all four parameters observed in the studied lakes were well within the validation ranges of the OC-CCI products^21,22^. Thus, we can say that optical properties of the World largest lakes allow to use the OC-CCI products in the trend analysis attempted by us.

In situ data from the studied lakes is relatively scarce, especially for parameters like CDOM and backscattering. This was the main reason of using remote sensing products and carrying out this study. Nevertheless, we were able to find in situ data from the Laurentian Great Lakes^23–29^, Lake Malawi^30–32^, Lake Tanganyika^33^, the Caspian Sea^34^, Lake Baikal^35,36^, and Lake Victoria^37^. Comparing the OC-CCI product values with the in situ results from published literature shows that the OC-CCI products are a reliable source of information for the type of analysis undertaken in this study.

Many studied lakes showed increase in lake CDOM in the last decade even if the overall trend for the 1997–2023 time period was negative. Increase in CDOM concentration is called browning. Current scientific literature suggests that browning is a phenomenon that can be found only in some Boreal/Nordic lakes^16,17,38^. Browning has positive effect on lake ecosystems as it protects aquatic biota against the damaging effects of UV radiation. However, increased absorption of light by CDOM decreases the amount of light available for primary production. Especially, because photosynthetic pigments, like chlorophyll-a, use blue light that is strongly absorbed by CDOM. Browning makes the euphotic depth shallower and radiative heating of water takes place in a thinner top layer of lakes. Rapid warming of lake surface waters has been observed globally^4^. However, they were not able to fully explain the lake temperature increase. Maybe browning of lake waters, that has taken place also in the World largest lakes during the last decade, contributes to this increase?

Stronger warming of thinner surface layer caused by browning also causes stronger thermal stratification and alters physical processes taking place in lakes^9,19^. Browning does also affect fish and fisheries^16,39^ and is increasing the costs in drinking water industry as removing the carbon requires higher quantities of costly chemicals^40^. Moreover, higher carbon concentration means that chlorinating (typical last stage of the treatment) creates more carcinogenic compounds^41^ in the process.

The studied lakes contain 60% of inland water volume. Consequently, even a small increase in their carbon content increases the global lake carbon stock significantly. Toming et al.^42^ found that the DOC stock of the Caspian Sea is 421 Tg while the global lake carbon stock is 729 Tg. The results of this study (Fig. 1) show that CDOM concentration in the Caspian Sea doubled during some periods of the 1997–2023. This implies that the closely correlated DOC amount also doubled during the same period. Consequently, the fluctuations in the Caspian Sea organic carbon stock may be as large as the global carbon stock of all other (small) lakes on Earth (which were not included in this study). The Caspian Sea gets water from more than hundred rivers which watersheds cover six climatic zones^43^ and experiences also variations in water level. Consequently, it is a very complex system, but monitoring the changes in CDOM/DOC with remote sensing products like the OC-CCI *a*_dg_(412) will allow to bring more insight into the processes taking place in the Caspian Sea.

Minor and Brinkley^44^ have shown that there was a minimum in the Laurentian Great Lakes water level around 2009–2010 followed by steep increase over the last decade. It has been suggested that there may be a relationship between the increasing water level and its CDOM content^11^. NOAA has Great Lakes Environmental Research Laboratory (GLERL) database that contains also lake level data^45^. We plotted the water level data in the Great Laurentian Lakes CDOM time series figures (Fig. 1). And indeed, the behaviour of monthly mean a_dg_(412) values matches well with the lake level data from the NOAA-GLERL database i.e. when the lake level had peaks in the late 1990s and late 2010s then the same happened with CDOM (Lakes Superior, Michigan, and Huron). The downstream lakes (Erie and Ontario) have more stable water level and no big variation in CDOM. Lake Erie has quite big variability between monthly means of all studied parameters (Fig. 1). It seems that the lake depends much more on the autochthonous processes (e.g. algal blooms) rather than the amount of inflow from the catchment area. The NOAA-GLERL data shows decrease in water level of Lakes Superior, Michigan and Huron in the last 2–3 years (Fig. 1)^45^. Thus, we may expect that the a_dg_(412) absorption will go down as well.

There is another substance, besides the CDOM and detrital matter (which absorption is summarised in the a_dg_(412)), that makes water brown—colloidal iron associated to DOC^46,47^. Rising iron concentrations have been observed in some landscapes ^48^. It has been noted that this rise in iron is not proportional to the rise in DOC ^49^. However, it has been found^50,51^ that iron has only minor effect on our ability to map lake CDOM with remote sensing. This allows to assume that the a_dg_(412) estimates made for the World largest lakes are not too much affected by the colloidal iron.

It has to be stressed that the browning of the World largest lakes found in this study is relative i.e. the absolute a_dg_(412) values are still low compared to many smaller lakes and even to some seas. The maximum values of the a_dg_(412) in most of the studied lakes remained below 0.3 m^−1^ and reached twice as high values only in Lakes Erie and Victoria. For the comparison, in the Baltic Sea the a_dg_(412) values vary between 0.26 m^−1^ (in the central part of the sea) and 20 m^−1^, while they may reach 60 m^−1^ in dark brown lakes connected with wetlands^52–54^.

In lakes CDOM is usually in good correlation with DOC and more than 90% of the total organic carbon is in the form of DOC^55^. On the other hand, CO_2_ saturation in lakes is closely linked to DOC ^56^. Consequently, our CDOM (a_dg_(412)) results give indication about the DOC/TOC/pCO_2_ behaviour in the World largest lakes, incorporating 60% of the global lake volume, over the 1997–2023 period. Lakes, as the lowest points in landscape, are also integrators of changes happening in land ecosystems^57^. Therefore, our study may provide also insight into climate change related processes happening on land.

A recent study ^58^ shows that unlike in many other parts of the world lake CDOM is going down in China. However, it has been observed both in Europe and North America that acid rain and other chemical pollution made lake waters more transparent and blueish in the last decades of the previous century. These regional effects were clearly seen also in remote sensing data decades after the polluting factories were shut down^59^. Thus, the decrease in lake CDOM in China may be due to local and regional processes rather than global processes.

Some literature suggests that phytoplankton blooms are intensifying in lakes globally^7,8^. However, the results of this study show that Chl-a increased only in a few of the World largest lakes (see Fig. 2). In several large lakes (e.g. Michigan, Huron, Victoria) the phytoplankton biomass has gone significantly down. Fang et al.^60^ studied all World lakes larger than 1 km^2^ in size. The results showed that the bloom frequency and area increased in two thirds of world lakes and decreases in one third. Thus, the increase in global temperature and CO_2_, that should favour increasing biomass in lakes, does not have such straightforward effect on lake ecosystems. The trends found in this study are consistent with the earlier studies that are based on in situ data, especially in regions where more in situ data is available e.g. in the Laurentian Great Lakes^5,29^. An earlier study^35^ for Lake Baikal showed that mean Chl-*a* increased from 0.82 to 1.20 mg/m^3^ over the 1977–2003 time period. Our study showed that the mean Chl-*a* for 2003 (the last year of the above mentioned in situ study) was 1.23 mg/m^3^ (i.e. the values of the OC-CCI data match very well with in situ data) and that the slightly increasing trend continues.

Sayers et al.^61^ studied primary production of eleven World largest lakes based on MODIS satellite products for 2003–2018 period. The primary production was calculated using some of the parameters used also in this study (e.g. Chl-a). Unfortunately, Sayers et al.^61^ published only annual primary productions for each lake that were calculated using some satellite products and some empirical constants. Therefore, it is not possible to make direct comparisons (e.g. Chl-a trends) between the two studies (the two different databases used).

The b_b_555 describes backscattering by all particles, including phytoplankton. Most of the particulate matter in large and deep lakes is phytoplankton as mineral particles (usually transported to the lakes by rivers) settle to the bottom quickly and cannot be resuspended. Thus, the increase or decrease in Chl-*a* should be reflected in the b_b_555 unless there is a change in the phytoplankton community. Small cells scatter light more effectively than large cells^62,63^. Large cells, on the other hand, tend to contain larger amount of Chl-*a*^64,65^. Consequently, different behaviour of Chl-a and b_b_555 trends is an indicator that there may be shift in phytoplankton community composition. The Fig. 2 shows that there may have been shifts in phytoplankton community in Lakes Michigan Huron and Victoria have taken place in the beginning of this millennium when the relationship between b_b_555 and Chl-a changed. This remote sensing based result needs further investigation i.e. detailed analysis on the phytoplankton communities in these lakes.

The studied lakes have relatively clear water which properties are mostly defined by phytoplankton biomass. Therefore, it is not surprising that the trends in K_d_(490) (Fig. 3 and Table 1) are similar to the trends in Chl-a (Fig. 2). On the other hand, the K_d_(490) is an important parameter to estimate primary production in lakes^66^ or calculate radiative heating^34^. Thus, it was important to study the numerical values of K_d_(490) in the World largest lakes and possible trends in the values. This information could bring us closer to understanding of the currently unexplained part in warming of lakes^67^.

The results of the study show that remote sensing data, and especially the long time series, allows to get interesting insight into the behaviour of lake ecosystems in time. The results of our study provide more food for thoughts to limnologists who model reaction of lake ecosystems on climate change and other pressures.

## Methods

### Satellite data

The data used in this study was downloaded from the freely available ESA OC-CCI data portal https://www.oceancolour.org/. The products can be downloaded via ftp, analysed in GIS portal, or accessed through different frameworks. The ESA OC-CCI monthly mean products (v6) with 4 km spatial resolution^68^ were used in this study. A detailed description of all the products is provided in the Product User Guide https://esa-oceancolour-cci.org/documents-list. The OC-CCI dataset is created from SeaWiFS, MODIS, MERIS, VIIRS, and OLCI satellite data to look like a continuous OLCI data series. The OC-CCI team has performed all the necessary calculations to take into account differences in spectral bands of the satellites (i.e. performed band shifts), sensitivities and spatial resolutions of the sensors, etc. The reflectance product used (v6) is like a long time series (since autumn 1997) of OLCI data (that actually was launched in 2016). The algorithms used to obtain the concentrations of optically active substances (e.g. Chl-a) are based on optical water type classification, different algorithms for each water type and algorithm blending. The inherent optical water properties (i.e. *a*_dg_, and *b*_bp_) have been calculated using the QAA approach^69^.

The lakes included in this study were the Caspian Sea, L. Baikal, L. Tanganyika, L. Superior, L. Michigan, L. Huron, L. Erie, L. Ontario, L. Malawi, L. Victoria and the Great Bear Lake. Monthly mean products covering 1997–2023 time period were used. The original OC-CCI flagging was used in this study. Meaning that land, cloud, ice and other masks were applied by the OC-CCI team. However, it was identified visually that some areas, for which OC-CCI data exist, are sometimes optically shallow (bottom is seen). Shallow bays, like Garabogaz and the Gorgan Gulf in the Caspian Sea, were excluded from the analysis. In some cases, it was obvious that formation of ice in autumn or remaining ice in spring were not properly masked out in the OC-CCI products as the mean values for the entire lake differed dramatically from the neighbouring months. These data points were eliminated from the further analysis.

Four parameters from the OC-CCI database were analysed: light absorption coefficient due to detrital and dissolved matter at 412 nm (*a*_dg_(412)), concentration of chlorophyll-a (Chl-a), light backscattering coefficient at 555 nm (*b*_bp_(555)), and diffuse attenuation coefficient of downwelling light at 490 nm (*K*_d_(490)). The OC-CCI uses a blended combination of algorithms for estimating Chl-a concentration (see details in the product user guide https://docs.pml.space/share/s/okB2fOuPT7Cj2r4C5sppDg). The *b*_bp_(555), *a*_dg_(412), and *K*_d_(490) are calculated using the QAA algorithm^69^.

The monthly mean values for each lake calculated from all available pixels are shown in the Figs. 1, 2, 3. Some of the lakes freeze over for shorter or longer periods. Some of the lakes have floating vegetation that covers extensive areas. Consequently, there are less monthly mean values in time series of lakes that freeze over and the number of pixels that are used to calculate the monthly means is not constant for each lake.

### Trend analysis

Trend analysis was performed using seasonal Mann–Kendall test^70^ improved by Hirsch et al.^71^ to take into account seasonality in the data. The Seasonal Mann–Kendall test is widely used for detecting trends in hydrometeorological data, such as temperature, precipitation, water quality, etc. (see^72^ and references therein). While the Mann–Kendall test is showing whether there is a trend or not, the magnitude or the trend was indicates by the number of the *z*-stats. The positive or negative number of *z*-stats shows increasing or decreasing trend, correspondingly. The analysis was performed using Real Statistics Resource Pack. A 95% confidence level was used in order to determine the trend significance.

## Data Availability

The data used in this study was downloaded from the freely available ESA OC-CCI data portal oceancolour.org. Version 6 data (Sathyendranath et al. 2021a) were used. A detailed description of all the products is provided in the Product User Guide https://esa-oceancolour-cci.org/documents-list. Trend analysis was performed using seasonal Mann–Kendall test using in freely Real Statistics Resource Pack.
